# The Role and Therapeutic Perspectives of Sirtuin 3 in Cancer Metabolism Reprogramming, Metastasis, and Chemoresistance

**DOI:** 10.3389/fonc.2022.910963

**Published:** 2022-06-27

**Authors:** QingYi Zhao, Jing Zhou, Feng Li, Sen Guo, Liang Zhang, Jing Li, Qin Qi, Yin Shi

**Affiliations:** ^1^ Department of Acupuncture and Moxibustion, Yueyang Hospital of Integrated Traditional Chinese and Western Medicine, Shanghai University of Traditional Chinese Medicine, Shanghai, China; ^2^ Department of Acupuncture and Moxibustion, Shanghai TCM-Integrated Hospital, Shanghai University of Traditional Chinese Medicine, Shanghai, China; ^3^ Department of Acupuncture and Moxibustion, Shanghai Municipal Hospital of Traditional Chinese Medicine, Shanghai University of Traditional Chinese Medicine, Shanghai, China; ^4^ Outpatient Department, Shanghai Research Institute of Acupuncture and Meridian, Shanghai, China

**Keywords:** sirtuin 3, cancer, metabolism reprogramming, metastasis, chemoresistance, activator, inhibitor, therapy

## Abstract

Sirtuin 3 (SIRT3), the nicotinamide adenine dinucleotide (NAD^+^)-dependent deacetylase, acts as a metabolic modulator mainly located in mitochondria *via* regulating the process of the relevant biochemical processes by targeting crucial mediators. Recently, owing to its dual role in cancer, SIRT3 has attracted extensive attention. Cancer cells have different metabolic patterns from normal cells, and SIRT3-mediated metabolism reprogramming could be critical in the cancer context, which is closely related to the mechanism of metabolism reprogramming, metastasis, and chemoresistance in tumor cells. Therefore, it is crucial to elucidate the relevant pathological mechanisms and take appropriate countermeasures for the progression of clinical strategies to inhibit the development of cancer. In this review, existing available data on the regulation of cancer metabolism reprogramming, metastasis, and chemoresistance progression of SIRT3 are detailed, as well as the status quo of SIRT3 small molecule modulators is updated in the application of cancer therapy, aiming to highlight strategies directly targeting SIRT3-mediated tumor-suppressing and tumor-promoting, and provide new approaches for therapy application. Furthermore, we offer an effective evidence-based basis for the evolvement of potential personalized therapy management strategies for SIRT3 in cancer settings.

## Introduction

Sirtuins are nicotinamide adenine dinucleotide (NAD^+^)-dependent class III histone deacetylases (HDACs) with homology to the *saccharomyces cerevisiae* silent information regulator 2 (Sir2) (1). Different from traditional HDACs, sirtuins deacetylate a series of substrates (2, 3). Moreover, as metabolic sensors, sirtuins connect their enzymatic activity with redox status and cellular energy through the co-factor NAD^+^ (1, 4). There are seven sirtuins, namely sirtuin 1-7 (SIRT1-7), that have been found in mammals. All sirtuins depend on NAD^+^, but they differ in location, types of post-translational modifications, and substrate affinity. Among these, SIRT3 has attracted much attention, as it exhibits the most powerful activity of mitochondrial deacetylase. SIRT3 could regulate a variety of mitochondrial proteins’ acetylation levels, such as metabolic enzymes and transcription factors, thereby playing a vital role in all kinds of cellular activities (5). Dysregulation of SIRT3 in cellular pathways may promote carcinogenesis and resistance to chemotherapy, which has been demonstrated in previous studies (6, 7). Thus, as a potential marker, SIRT3 expression could be identified in patients susceptible to metastasis or drug-resistant disease when cancer is initially diagnosed. Cancer remains a major health problem worldwide, and cancer metastasis is a principal consideration of cancer-related fatalities. In 2011, Hanahan and Weinberg summarized ten hallmarks of cancerous cells in *Cell* that include resisting cell death, enhancing invasion and metastasis, maintaining proliferative signaling, dysregulation of cellular energy, inducing angiogenesis, limitless replicative potential, evading immune destruction, gene instability and mutation, escaping growth inhibitors, and tumor-promoting inflammation (8). In fact, these ten cancer phenotypic features that differ from normal cells reflect altered metabolism or metabolism reprogramming of tumor cells. Despite recent advancements in cancer treatment, the chemotherapy resistance of neoplastic cells directly affects the prognosis of patients. Therefore, novel target-based avenues to enhance treatment effectiveness need to be explored.

Currently, owing to its dual role in cancer, SIRT3 has attracted lots of attention from researchers, particularly in the terms of regulating the metabolism reprogramming of tumor cells, involving cancer metastasis, drug resistance, and other aspects. Although reviews on the dual role of SIRT3 in cancer have been summarized and published, there is currently no systematic review on cancer metastasis and drug resistance about the role of SIRT3. In this review, we mainly pay close attention to how SIRT3 plays a dual role in regulating the cancer metabolism reprogramming, metastasis, and chemoresistance, as well as further summarize the SIRT3 small molecule modulators in the application of cancer therapy. Furthermore, we provide evidence to support the development of potential personalized therapy strategies for SIRT3 in cancer settings.

## The Location and Structure of SIRT3

**Figure 1:**
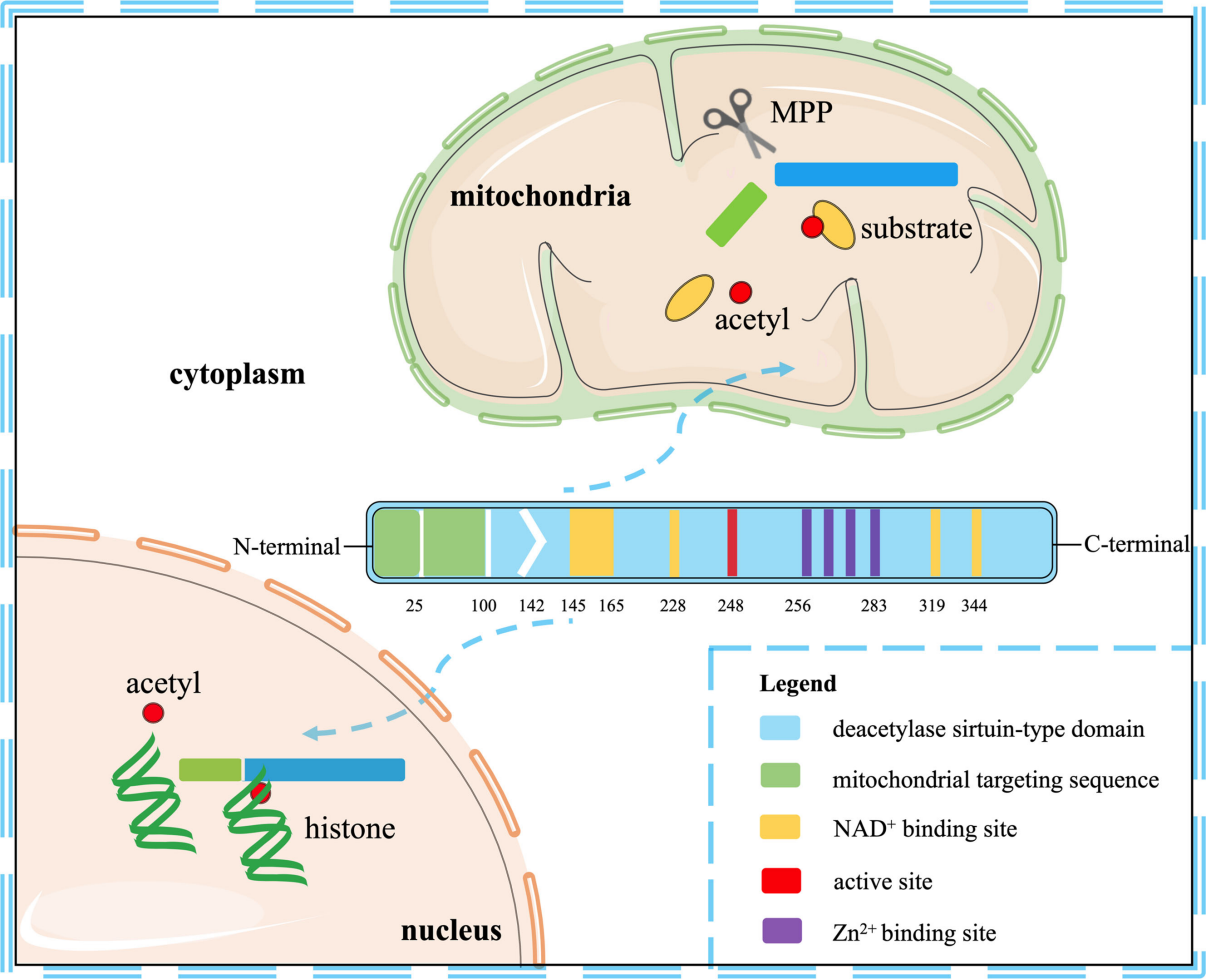
The location and structural composition of SIRT3. The conserved enzymatic core of SIRT3 contains an active site, four Zn^2+^ sites, four NAD^+^ sites, and a mitochondrial targeting sequence. SIRT3 is directed to the mitochondria through the targeting sequence, where it is cleaved by the mitochondrial MPP to produce mature proteins. As a deacetylase, it is primarily localized to mitochondria, but also shows histone deacetylation activity in the nucleus.

Seven mammalian sirtuins (SIRT1-7) have different flanking N-terminal and C-terminal extensions, which are in charge of the subcellular location of sirtuins. As a typical sirtuin, SIRT3 is a mitochondrial protein, although SIRT3 has also been confirmed to shift from the nucleus to the mitochondria under stress conditions (9, 10). Although all types of sirtuins are variable in length and sequence, they all have a catalytic core domain that is highly conserved with about 275 amino acids (11). The highly conserved enzymatic core contained in SIRT3 has two main domains, one is a large Rossmann fold domain and the other is a small zinc-binding domain. The large Rossmann fold domain shows the classic features of NAD^+^ binding sites and the specific features that recognize nicotinamide or ribose groups. The small zinc-binding domain can be served as an appealing and latent binding site for sirtuin inhibitors. The cleft between the two domains could be used as a docking site of acetylation substrates, where the NAD^+^ and peptide substrates containing acetyl-lysine enter and bind to the enzymes for deacetylation (12). In humans, SIRT3 is expressed as a full-length 44-kDa protein and is targeted to the cellular mitochondria proteins through its N-terminal location sequence. SIRT3 is expressed firstly in the cytoplasm as an inactive precursor of 399-amino acid (44-kDa). During the cellular stress, SIRT3 translocates to the mitochondrial matrix and is activated and stimulated by the mitochondrial matrix processing peptidase (MPP), which yields a comprising 257-amino acid residues (28-kDa) the short active form of SIRT3 (10, 13). Mitochondrial transport mainly relies on the N-terminal 100 amino acids, among which residues 1-25 play a vital role in mitochondrial targeting and proteolytic processing (13). Most studies support the localization of SIRT3 in mitochondria, but some studies have proved that SIRT3 may also exist in the nucleus, and it usually shows histone deacetylation activity and translocates to the mitochondria under stress conditions (10, 14). Hence, SIRT3 may autoregulate itself between the nucleus and the mitochondria. Despite the controversy, it can be concluded that SIRT3 mainly exerts a key role in the cellular mitochondria. The location and structural composition of SIRT3 is shown in **Figure 1**.

## SIRT3 Deacetylation

The SIRT3 protein is mainly expressed in mitochondria-abundant tissues with metabolic activity, including the kidney, liver, brain, heart, and brown adipose tissue (15). As an important deacetylase in mitochondria, SIRT3 affects mitochondrial metabolism and function by modulating the acetylation level of the critical enzymes in mitochondrial metabolism to maintain the normal metabolic balance of cells (16). Mitochondria metabolism and function are highly regulated by protein acetylation. As a mitochondrial important and classic regulatory mechanism, acetylation could alter the protein-protein interactions network, affect complex stability, decide the location in mitochondria, and modulate enzyme activity. Acetylated proteins are richly expressed in the cells, such as the nucleus, mitochondria, and cytoplasm. Although mitochondrial proteins are highly acetylated leading to mitochondrial dysfunction, enzymes that regulate acetylation are rarely reported (17, 18). To be precise, the acetylation process is a biochemical reaction in which the acetyl group of acetyl-CoA is transferred to the amino acid residue of proteins with the help of acetyltransferase (19, 20). Deacetylation modification depends on the catalytic deacetylation function of deacetylase and serves as the reverse conversion of the acetylation reaction. The reaction process of NAD^+^-dependent SIRT3 deacetylation is rough as follows. The acetylated substrate and the NAD^+^ co-substrate combined with SIRT3 in the first place. Then, the acetyl group transfers from substrate to ADP-ribose moiety of NAD^+^, resulting in the production of bicyclic intermediates, which ultimately generate the deacetylated protein. Translocation in cells or conformational changes will occur after substrate deacetylation, which adjusts the activity of the substrate enzyme and regulates the reaction process involved by the enzyme (21, 22).

SIRT3 is mainly located in the mitochondria and functions as a vital role in regulating the glucose metabolism, tricarboxylic acid (TCA) cycle, oxidative phosphorylation (OXPHOS), reactive oxygen species (ROS) detoxification, electron transport chain (ETC), fatty acid (FA) metabolism, mitochondrial unfolded protein response (UPR^mt^), autophagy, etc. (23–25). SIRT3 could directly deacetylate pyruvate dehydrogenase (PDH) and glutamate oxaloacetate transaminase 2 (GOT2) to regulate glucose metabolism (26). As a multienzyme complex, PDH converts pyruvate to acetyl-Co, and the K336 site of its core subunit pyruvate dehydrogenase E1α (PDHA1) may be served as the potential deacetylation site of SIRT3 (27). The acetylation of GOT2 3K (K159, K185, K404) sites could improve the ability of the malate-aspartate shuttle, but GOT2 deacetylation through SIRT3 shows an inhibited function (28). In addition, SIRT3 could regulate the TCA cycle *via* deacetylating isocitrate dehydrogenase 2 (IDH2) and glutamate dehydrogenase (GDH). IDH2 is the rate-limiting enzyme of the TCA cycle to some certain extent in the case of SIRT3 low expression (29). The acetylation level of IDH2 at K413 and K360 sites is increased, and the decreased enzyme activity leads to the inability to participate in electronic transmission, thus obstructing the TCA cycle (30, 31). Similarly, SIRT3 deacetylates GDH and regulates the TCA cycle to promote amino acid oxidation, as well as one of the GDH acetylation sites, K527, has been reported (32, 33). Mitochondria produce ATP through OXPHOS, and SIRT3 deacetylates ETC subunits, such as complexes I–III and ATP synthase, to maintain cellular ATP levels (34, 35). Succinate dehydrogenase (SDH) is an enzyme that exists between the TCA cycle and the ETC (36). SIRT3 could directly deacetylate SDH A subunit, and the K179 site may be a potential deacetylation site (31). It is well known that the production of ROS is closely related to OXPHOS transferred electrons and mitochondrial energy metabolism, and SIRT3 can stabilize ROS levels by deacetylation of ETC proteins (37, 38). In addition, SIRT3 can directly deacetylate and activate antioxidant enzymes, such as IDH2 and superoxide dismutase 2 (SOD2), to protect cells from ROS (39, 40). SIRT3 can also deacetylate transcription factor forkhead box 3α (FOXO3α) to regulate the expression of antioxidant enzymes, and deacetylated sites of FOXO3α are mainly located at K271 and K290 (41, 42). Thus, activation of SIRT3 may contribute to ROS detoxification, thus avoiding oxidative stress damage. Moreover, SIRT3 plays an important role in regulating FA metabolism by deacetylation of long-chain-specific acyl-CoA dehydrogenase (LCAD) at K42, K318, and K322 sites (22, 43). In other aspects, as the primary coordinator of UPR^mt^, SIRT3 not only maintains protein homeostasis but also deacetylates the autophagy-related protein to repair defective autophagy, which is important for cell function and survival (44, 45). Consequently, as the key site of energy metabolism, mitochondria could regulate cellular metabolism and intracellular signal transduction to maintain homeostasis, and their imbalance indicates the arrival of diseases (46). Acting as a modulator of cell metabolism, SIRT3 displays tumor-suppressing and tumor-promoting features in cancer by regulating the acetylation level of multiple mitochondrial proteins (47). Although the most basic biological function of SIRT3 is the deacetylation of its substrate, cancer cell pathophysiology, metabolism reprogramming, metastasis, and chemoresistance are dependent on the regulation of SIRT3 deacetylation.

## Regulation of Cancer Metabolism Reprogramming by SIRT3

**Figure 2:**
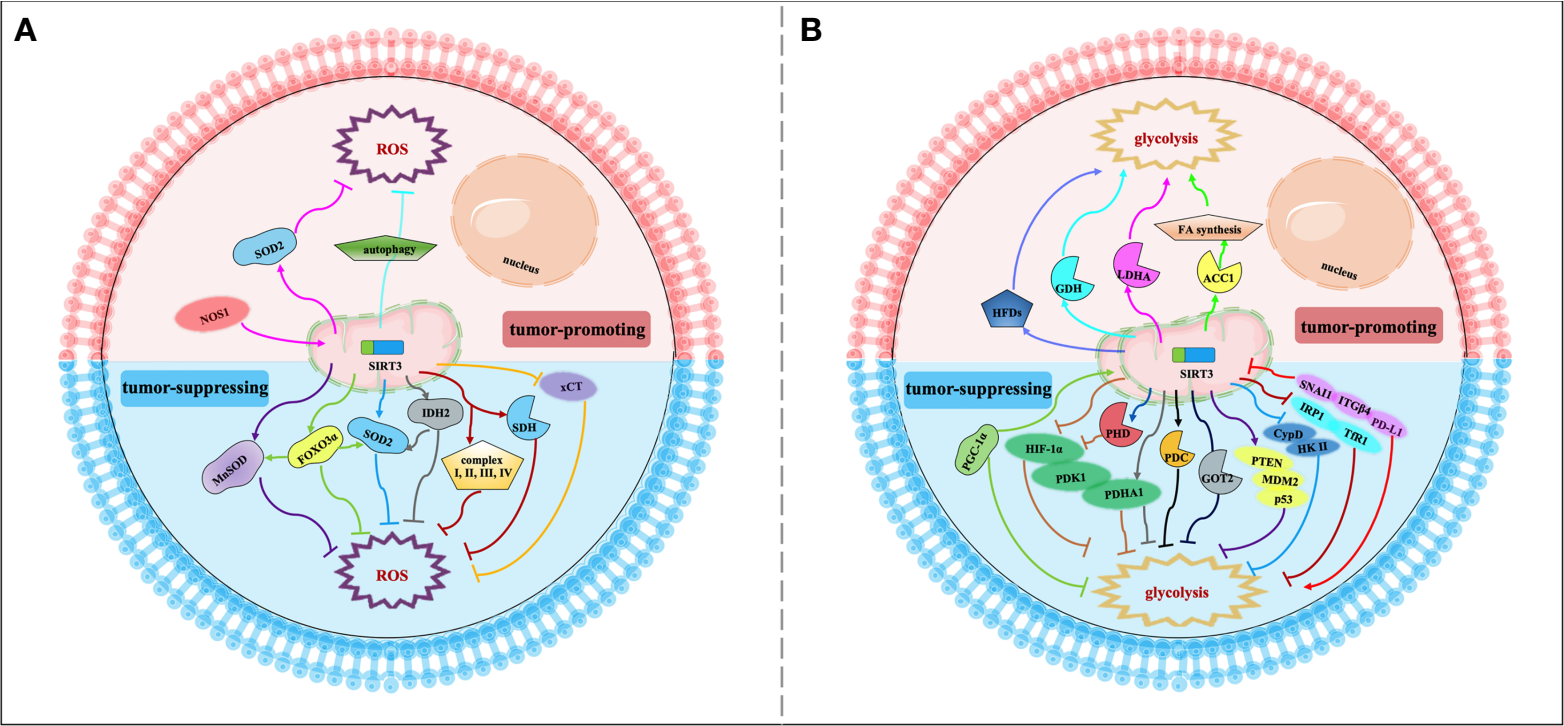
Signaling functions of SIRT3 relevant to cancer metabolism reprogramming. **(A)** SIRT3 regulates cancer metabolism reprogramming by modulating ROS levels. As a tumor-suppressing protein, SIRT3 could deacetylate and modulate the activity of a series of enzymes and transcription factors such as SOD2, MnSOD, FOXO3α, IDH2, SDH, and xCT to decrease the ROS levels and regulate tumor metabolism reprogramming. As a tumor-promoting protein, SIRT3 could regulate the NOS1/SIRT3/SOD2 pathway or mitochondrial autophagy to keep ROS at an appropriate level to promote tumorigenesis and prevent apoptosis. **(B)** SIRT3 regulates cancer metabolism reprogramming by modulating tumor cell glycolysis. As a tumor-suppressing protein, SIRT3 not only deacetylates and regulates the activity of various enzymes and proteins including HIF-1α, PHD, PDHA1, GOT2, CypD, IRP1, and PGC-1α, but also affects some signaling pathways such as HIF-1α/PDK1/PDHA, PTEN/MDM2/p53 pathway, CypD/HK II pathway, IRP1/TfR1 pathway, and PD-L1/ITGβ4/SNAI1 pathway, thereby inhibiting glycolysis and regulating tumor metabolism reprogramming. As a tumor-promoting protein, SIRT3 could deacetylate and activate ACC1, LDHA, GDH, and HFDs to promote glycolysis and increase the occurrence of cancer.

The most striking feature of metabolism reprogramming of tumor cells is the “Warburg effect”, which is that most cancer cells are more dependent on aerobic glycolysis than OXPHOS. Glycolysis could provide a rapid energy supply and several beneficial conditions for the genesis and progress of the tumor microenvironment. To meet the needs of continued growth and proliferation, tumor cells provide themselves with more material and energy through metabolism reprogramming (48). The signaling functions of SIRT3 relevant to cancer metabolism reprogramming are shown in **Figure 2** and **Table 1**.

**Table 1:** Regulation of cancer metabolism reprogramming by SIRT3.

Role of SIRT3	Targets/Pathways	Cells type of the experiment	Effect of SIRT3 action	References
Tumor-suppressing	SOD2	Human uterine cervical cancer cell lines (HeLa), HEK293T cells	Reduce ROS levels	(49)
	MnSOD	Human colorectal carcinoma cell lines (HCT116), MEFs	Decrease ROS production	(50)
	FOXO3α	Human colorectal carcinoma cell lines (HCT116), Cos-7 cells	Protect cells from oxidative stress	(42, 51)
	IDH2	HEK293T cells, MEFs	Decrease ROS production	(29)
	SDH	Human chronic myelogenous leukemia cells lines (K562)	Reduce ROS production	(52)
	xCT	Human breast cancer cell lines (MCF-7, MDA-MB-231, Hs-578t), HEK293T cells	Balance the ROS levels to protect cancer cells from glucose-deprivation-induced cell death	(53)
	HIF-1α	Breast cancer cell lines (MCF7, T47D, CAMA1), gastric cancer cells (MGC-803), human pancreatic cancer cell lines (MIA PaCa-2, SW1990, BxPC-3, CFPAC-1, Capan-1), HEK293T cells, MEFs	Repress glycolysis and proliferation	(34, 54, 55)
	PHD	HEK293T cells, MEFs	Decrease HIF-1α tumor-promoting ability	(34)
	PDHA1	Human colorectal carcinoma cell lines (HCT116), lung cancer cell lines (H1299), human cholangiocarcinoma cell lines (HuCCT1, RBE, HCCC9810), HEK293T cells	Inhibit glycolysis and the “Warburg effect”	(26, 56)
	HIF-1α/PDK1/PDHA1	Human cholangiocarcinoma cell lines (HuCCT1, RBE, HCCC9810)	Inhibit the “Warburg effect”	(57)
	GOT2	Pancreatic cancer cell lines (Panc-1), HEK293T cells	Block the growth of pancreatic tumors	(28)
	PTEN/MDM2/p53	Human breast cancer cell lines (MDA-MB-231, MCF-7), human colorectal carcinoma cell lines (HCT-116, HT-29)	Stabilize p53 and inhibit glycolysis	(58)
	CypD	Human breast cancer cell lines (MDA-MB-231, MCF-7)	Inhibit breast carcinoma glycolysis	(59)
	IRP1	Human pancreatic ductal adenocarcinomas cell lines (8988T, Panc1), MEFs	Regulate cellular iron metabolism	(60)
	PGC-1α	Human breast cancer cell lines (MCF-7, MDA-MB-231)	Suppress glycolytic metabolism	(61)
	PD-L1/ITGβ4/SNAI1	Human uterine cervical cancer cell lines (Siha, HeLa, C33A, Me180, MS751, Caski)	SIRT3 is inhibited by PD-L1 to regulate glucose metabolism and provide energy for cancer metastasis	(62)
Tumor-promoting	NOS1/SIRT3/SOD2	Human colon cell lines (SW480, SW620)	Regulate ROS production properly and suppress apoptosis of colon cancer cells	(63)
	ACC1	Human cervical cancer cell lines (SiHa, C33a), human immortalized cervical squamous epithelial cell lines (H8)	Promote the reprogramming of FA synthesis	(64)
	LDHA	Human gastric cancer cell lines (MGC-803, HGC-27, SGC-7901, AGS), immortalized human gastric epithelial cell lines (GES-1)	Promote glycolysis and increase ATP production	(65)
	GDH	Diffuse large B-cell lymphoma cell lines (OCI-Ly1, OCI-Ly7)	Promote the TCA cycle	(66)

SIRT3 can modulate intracellular ROS levels to regulate tumor metabolism reprogramming (**Figure 2A**). Elevated oxidative stress caused by ROS is the most important feature of mitochondria reprogramming in cancer cells (67, 68). Cancer cells have higher levels of ROS compared with normal cells. ROS can oxidize specific intracellular chemical moieties to damage macromolecules, such as DNA, leading to genetic mutations and influencing the biochemical pathways of tumor cell proliferation and transformation (69). Although excessive accumulation of ROS levels may trigger cell death, a slight increase in ROS may benefit cell survival and growth. Thus, the role of ROS remains controversial (70, 71). SIRT3 could function as a tumor suppressor to limit ROS levels *via* the activation of antioxidant enzymes, such as superoxide dismutase (SODs). Dysregulation of the detoxification activity of antioxidant enzymes in tumors implies more oncogenic or therapeutic resistance phenotypes. As an antioxidant enzyme, SOD2 can be deacetylated by SIRT3 to remove ROS (49). SIRT3 can also target the mitochondrial enzyme manganese superoxide dismutase (MnSOD) to restore superoxide dismutase activity and decrease ROS production (50). SIRT3 could also deacetylate another important target, FOXO3α, to up-regulate antioxidant enzymes and protect cells from oxidative stress (42, 51). In addition, IDH2 can be directly deacetylated by SIRT3, thereby affecting SOD2-mediated mitochondrial ROS scavenging and cellular redox status (29). SIRT3 can also regulate the activity of complexes I-IV in the ETC to reduce ROS leakage from the complex, and improve the detoxification ability of oxidase, thus reducing ROS production (38, 50). For example, SIRT3 can catalyze the deacetylation of the SDH flavoprotein subunit, one of the Complex II subunits, and coordinate material oxidation and electron transport to reduce ROS production by modulating SDH activity (52). Recently, one study deserves attention that SIRT3 may play a protective role in the glucose-deprivation-induced cell death of breast cancer cells by regulating the xCT expression, also known as solute carrier family 7 member 11 (SLC7A11), to balance the ROS levels (53). In this regard, SIRT3 is considered a key scavenger of ROS in cells by playing a tumor-suppressing effect. On the contrary, SIRT3-mediated ROS removal may also contribute to the oncogenic effects. SIRT3 keeps ROS at an appropriate level to maintain proliferative and aggressive phenotypes that promote tumorigenesis and prevent apoptosis (72). For instance, mitochondrial neuronal nitric oxide synthase (nNOS, NOS1) could increase SIRT3 activity and thereby activating SOD2 to regulate ROS production properly, thus, suppressing apoptosis of colon cancer cells (63).

Similarly, SIRT3 may also regulate tumor metabolism reprogramming by modulating tumor cell glycolysis (**Figure 2B**). As a tumor suppressor, downregulation of SIRT3 may promote a Warburg phenotype (73). In breast cancer and gastric cancer, the loss of SIRT3 promotes ROS production, leading to hypoxia-inducible factor-1α (HIF-1α) stabilization. HIF-1α is a transcription factor that controls glycolytic gene expression, and SIRT3 mediates metabolism reprogramming by destabilizing HIF-1α to affect the progression of cancer (34, 54). In this regard, profilin1 (Pfn1) played an anti-cancer role in pancreatic cancer by improving the SIRT3 expression, resulting in the destabilization of HIF-1α to inhibit glycolytic (55). SIRT3 can also directly deacetylate prolyl hydroxylase (PHD) to regulate HIF-1α, thereby decreasing the stability of HIF-1α to inhibit glycolysis (34). In addition, SIRT3 could deacetylate and activate pyruvate dehydrogenase complex (PDC) and related component enzyme PDHA1 to inhibit glycolysis and decrease the “Warburg effect” (26, 56). Interestingly, SIRT3 inhibits the cholangiocarcinoma progression *via* inhibiting the “Warburg effect” through the HIF-1α/pyruvate dehydrogenase kinase 1 (PDK1)/PDHA1 downstream signaling pathway (57). In addition, SIRT3 plays a potential role in the deacetylation of GOT2. SIRT3 impairs the protein association between GOT2 and malate dehydrogenase 2 (MDH2), thereby inhibiting the activity of the malate–aspartate shuttle. As a restriction enzyme that regulates the glycolysis process, GOT2 is deacetylated by SIRT3, thus obstructing the transport of cytosolic nicotinamide adenine dinucleotide hydrogen (NADH) into the mitochondria to achieve the purpose of blocking the growth of pancreatic tumors (28). p53 is one of the tumor suppressors that slows down glycolysis and promotes OXPHOS (74). SIRT3 could inhibit glycolysis *via* stabilizing p53 in cancer cells, which was related to the phosphatase and tensin homolog (PTEN) and mouse double minute 2 (MDM2) (58). SIRT3 deacetylates and inactivates cyclophilin D (CypD) to impact the contact of the lactate metabolic enzyme hexokinase II (HK II) with mitochondria, further inhibiting breast carcinoma glycolysis (59). Besides, SIRT3 inhibits the aggregation of iron regulatory protein 1 (IRP1), and further reduces the transferrin receptor (TfR1) expression, thus, regulating cellular iron metabolism to affect the proliferation of pancreatic cancer cells (60). Peroxisome proliferator-activated receptor γ coactivator 1α (PGC-1α) regulates the cell survival and proliferation of cancer cells by controlling mitochondrial biogenesis and cellular metabolism. PGC-1α could suppress glycolytic metabolism by activating SIRT3 to moderate breast cancer cell proliferation (61). As a downstream effector factor of programmed death-ligand 1 (PD-L1), tumor suppressor SIRT3 is inhibited by PD-L1 and participates in integrin β4 (ITGB4)/snail family transcriptional repressor 1 (SNAI1) signaling pathway to regulate glucose metabolism, which provides energy to promote metastasis in cervical cancer (62). A recent study has shown that whey could target SIRT3 expression to induce metabolic dysfunctions and modulate the bioenergy characteristics of colon cancer cells (75). Of course, as a tumor promoter, SIRT3 also shows a dark side. In cervical cancer, SIRT3 significantly promotes the FA synthesis reprogramming by up-regulating acetyl-CoA carboxylase (ACC1) (64). In addition, as a key protein regulating anaerobic glycolysis, lactate dehydrogenase A (LDHA) could be deacetylated and activated by SIRT3 to promote glycolysis and increase ATP production in gastric cancer cells (65). Similarly, SIRT3 enhances the activity of GDH to promote the TCA cycle, thereby increasing the occurrence of lymphoma (66). Intriguingly, inhibition of SIRT3 expression may reduce the carcinogenic effect induced by high-fat diets (HFDs), suggesting that SIRT3 is served as an oncogene (76).

## Regulation of Cancer Metastasis by SIRT3

**Figure 3:**
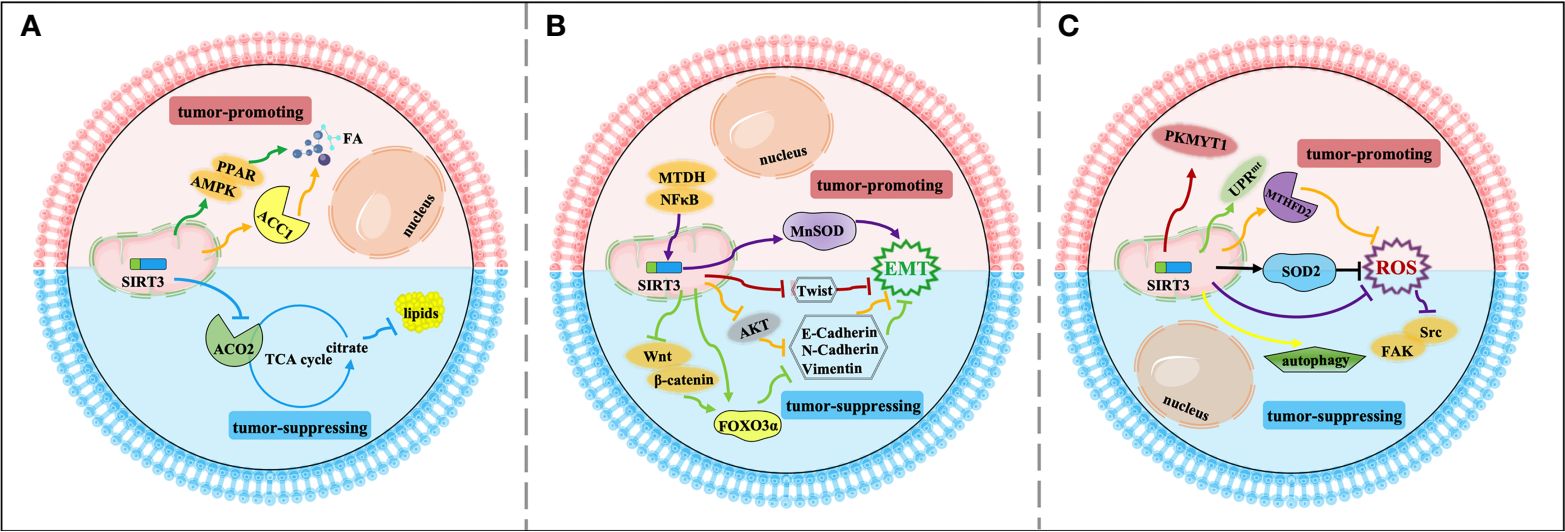
Signaling functions of SIRT3 relevant to cancer metastasis. **(A)** SIRT3 regulates cancer metastasis by modulating tumor metabolism reprogramming. As a tumor-suppressing protein, SIRT3 deacetylates and inactivates ACO2 to inhibit adipogenesis and cancer metastasis. As a tumor-promoting protein, SIRT3 deacetylates and activates ACC1 and the AMPK/PPAR pathway to mediate FA synthesis and promote cancer metastasis. **(B)** SIRT3 regulates cancer metastasis by modulating EMT. As a tumor-suppressing protein, SIRT3 up-regulates Twist and inhibits EMT-related markers such as E-cadherin, N-cadherin, and N-cadherin by affecting FOXO3α, AKT, and Wnt/β-catenin/FOXO3α pathway, thereby inhibiting EMT and cancer metastasis. As a tumor-promoting protein, SIRT3 regulates the NFκB/SIRT3/MnSOD pathway to promote EMT and cancer metastasis. **(C)** Other relevant mechanisms that SIRT3 participates in cancer metastasis. As a tumor-suppressing protein, SIRT3 regulates autophagy, and inhibits ROS production through SOD2 and the Src/FAK pathway, leading to suppression of cancer metastasis. As a tumor-promoting protein, SIRT3 deacetylates and regulates SOD2, MTHFD2, PKMYT1, and the SIRT3 axis of the UPR^mt^ to promote cancer metastasis.

The invasion and migration of tumor cells to surrounding tissues is the beginning and pivotal stage of cancer metastasis, which is a major principal consideration of cancer deaths. Regulation of metabolism reprogramming and EMT by SIRT3 can make a significant influence on cancer metastasis. Exploring the metabolic targets that drive invasive and metastatic cancers promises to be an effective therapeutic strategy. The signaling functions of SIRT3 relevant to cancer metastasis are shown in **Figure 3** and **Table 2**.

**Table 2:** Regulation of cancer metastasis by SIRT3.

Regulatory mechanism	Role of SIRT3	Targets/Pathways	Cells type of the experiment	Effect of SIRT3 action	References
Tumor metabolism reprogramming	Tumor-suppressing	ACO2	Prostate cancer cell lines (LNCaP, 22Rv1, PC-3, C4-2, VCaP, DU145, TRAMP-C2), HEK293T cells	Inhibit adipogenesis and the metastasis	(77)
	Tumor-promoting	ACC1	Human cervical cancer cell lines (SiHa, C33a), human immortalized cervical squamous epithelial cell lines (H8)	Mediate FA synthesis to promote the invasion and metastasis	(64)
		AMPK/PPAR	Cervical cancer cell lines (C33a, SiHa)	Enhance the ability to invade and migrate cancer cells	(78)
EMT	Tumor-suppressing	Wnt/β-catenin/FOXO3α	Human prostate cancer cell lines (C42B, DU145, PC3M), human small‐cell lung cancer cell lines (NCI‐H446, DMS114)	Inhibit EMT and migration	(79, 80)
		AKT	Human gall-bladder cancer cell lines (GBC-SD, EH-GB1, OCUG-1, NOZ)	Lead to ferroptosis and tumor suppression	(81)
		Twist	Human ovarian carcinoma cell lines (HO-8910, HO-8910PM)	Suppress the metastasis	(82)
	Tumor-promoting	NFκB/SIRT3/MnSOD	Triple-negative breast cancer cell lines (MDA-MB-231, BT-549), human breast cancer cell lines (MCF-7, MCF-10A), HEK-293T cells	Promote the metastasis and spread	(83)
Other relevant mechanisms	Tumor-suppressing	SOD2	Hepatocellular carcinoma cell lines (BEL7402, SMMC-7721, MHCC97H, HL-7702, MHCC97L, PLC/PRF/5, HepG2, Huh-1, HLE)	Inhibit ROS production to suppress metastasis	(84)
		Src/FAK	Human breast cancer cell lines (MCF10A cells, MDA-MB-231, LM2-4175, BoM-1833 cells)	Inhibit ROS production to suppress metastasis	(85)
	Tumor-promoting	SOD2	Human epithelial ovarian cancer cell lines (OVCA433, OVCA420, ES-2, NIH-OVCAR3)	Enhance ovarian cancer metastasis	(86)
		MTHFD2	Human colorectal carcinoma cell lines (HCT116), human uterine Cervical cancer cell lines (HeLa), HEK293T cells	Promote cellular redox balance and drive proliferation and migration	(87)
		PKMYT1	Ovarian epithelial cell lines (HOSEPiCs), ovarian cancer cell lines (PEO1, A2780, SKOV3, OVCAR3, 3AO, CAOV3)	Promote migration and invasion	(88)
		UPR^mt^	Breast cancer cell lines (MDA-MB-231, MDA-MB-361, MDA-MB-157, MCF7, MCF7R)	The activation of the SIRT3 axis of the UPR^mt^ is associated with metastasis	(89)

Firstly, metabolism reprogramming plays important role in tumor cell survival during the cancer metastasis process, which contributes to driving the successful formation of metastatic lesions (90). Studies have shown that changes in FA synthesis in cancer cells may offer selective advantages for the metastatic process (91, 92). The FA synthesis ability of tumor tissues with strong invasion ability is generally enhanced. Thus, for rapidly proliferating and strongly invasive tumor cells, there is high demand for FA (93). As a tumor suppressor, acetylation of aconitase (ACO2) could be reversibly regulated by SIRT3. Transcriptional repression of SIRT3 enhances the ACO2 activity to promote mitochondrial citrate synthesis and adipogenesis, further promoting the progression of aggressive prostate cancer to the bone (77). As a tumor promoter, SIRT3 promotes cervical cancer invasion and metastasis *via* up-regulating FA synthesis mediated through ACC1 (64). SIRT3 also activates the AMP-activated protein kinase (AMPK)/peroxisome proliferator-activated receptor (PPAR) pathway to effectively promote intracellular triglyceride synthesis. Increased FA synthesis of tumor cells enhances the invasion and infiltration *in vitro* and promotes the growth of primary tumors and distant metastases *in vivo*, which further enhances the ability of cervical cancer cells to invade and migrate (78, 94) (**Figure 3A**).

Moreover, SIRT3 may play a dual role in cancer metastasis by affecting epithelial-to-mesenchymal transition (EMT). EMT-mediated metastasis is when cancer cells spread from the primary tumor to adjacent tissues firstly, then spread and proliferate *via* the bloodstream and lymphatic system. From the perspective of mechanism, EMT regulates epithelial markers, mesenchymal markers, and transcriptional regulatory factors to play a dual role in cancer metastasis mainly manifested in down-regulation of E-cadherin and cytokeratin, up-regulation of N-cadherin, Vimentin, β-catenin, and Snail. SIRT3 plays a significant inhibitory role in the cancer metastasis process. SIRT3 regulates the EMT-related markers such as E-Cadherin, N-Cadherin, and Vimentin to suppress EMT, especially in prostate cancer and small‐cell lung cancer, which may be related to Wnt/β-catenin/FOXO3α pathway and protein kinase B (AKT, also known as PKB)-dependent ferroptosis (79–81). In ovarian cancer, as a key regulator of EMT, Twist is down-regulated by SIRT3 to suppress the metastasis of the tumor (82). Interestingly, SIRT3-mediated activation of MnSOD can promote EMT in cancer. SIRT3 could be mediated by metadherin (MTDH) to enhance its activity, and enhance EMT and invasion by participating in the NFκB/SIRT3/MnSOD pathway, ultimately promoting the metastasis and spread of triple-negative breast cancer (83) (**Figure 3B**).

Finally, in addition to regulating metabolism reprogramming and EMT, there are other potential mechanisms that SIRT3 participates in cancer metastasis. Down-regulation of SIRT3 promotes tumor metastasis ability, in which it plays a tumor suppressor role. ROS contributes to increased invasion and migration capacity, which is associated with the inhibition of SOD2 by mitochondrial Ca^2+^ uniporter (MCU)-dependent mitochondrial Ca^2+^ uptake (84). Moreover, down-regulation of SIRT3-mediated elevated ROS levels could promote the activation of the steroid receptor coactivator (Src)/focal adhesion kinase (FAK) signaling pathway to enhance cancer metastasis (85). SIRT3 also significantly promotes the expression of key proteins involved in autophagosome formation, thereby inducing proliferation and migration inhibition (95). However, as a tumor promoter, a recent study showed that SIRT3 could enhance ovarian cancer metastasis by rapidly up-regulating SOD2, which was a new piece of evidence of SIRT3’s role in promoting metastasis in cancer (86). SIRT3 can directly deacetylate the mitochondrial methylenetetrahydrofolate dehydrogenase/cyclohydrolase (MTHFD2) to promote cellular redox balance and drive colorectal cancer cell proliferation and diffusion (87). In ovarian cancer, SIRT3 enhances the ability of protein kinase membrane associated tyrosine/threonine 1 (PKMYT1) to promote cell migration and invasion, thus accelerating cancer metastasis (88). Furthermore, SIRT3 is a necessary component of the UPR^mt^ required for breast cancer metastasis to secondary organs (89, 96), which further enriches our information on the dual role of SIRT3 in the regulation of cancer metastasis (**Figure 3C**).

## Regulation of Cancer Chemoresistance by SIRT3

**Figure 4:**
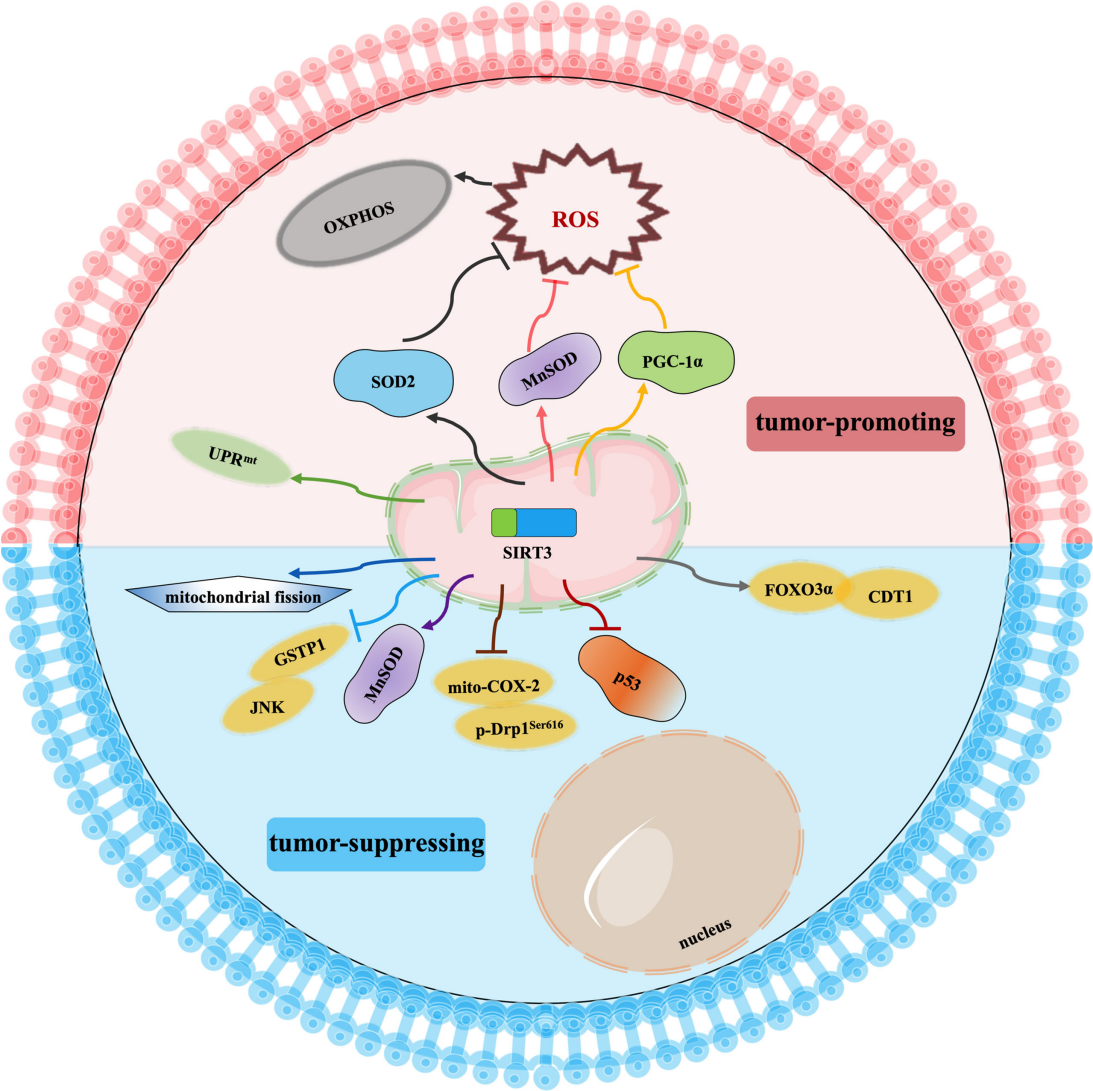
Signaling functions of SIRT3 relevant to cancer chemoresistance. As a tumor-suppressing protein, SIRT3 not only deacetylates MnSOD and p53 but also regulates the mito-COX-2/p-Drp1^Ser616^ pathway, SIRT3/GSTP1/JNK pathway, FOXO3α/CDT1 pathway, and mitochondrial fission to enhance chemotherapy drugs sensitivity of cancer. As a tumor-promoting protein, SIRT3 scavenges the oxidant products by deacetylating SOD2, MnSOD, and PGC-1α, and activates UPR^mt^ to promote chemotherapy drugs resistance to cancer.

Cancer drug resistance is the main factor leading to chemotherapy failure. Research on the mechanism of cancer chemoresistance is essential to establishing therapeutic strategies for drug-resistant tumors. Based on the dual role of SIRT3 in cancer, its involvement in cancer drug resistance is also bidirectional. The signaling functions of SIRT3 relevant to cancer chemoresistance are shown in **Figure 4** and **Table 3**.

**Table 3:** Regulation of cancer chemoresistance by SIRT3.

Role of SIRT3	Targets/Pathways	Cells type of the experiment	Effect of SIRT3 action	References
Tumor-suppressing	mito-COX-2/p-Drp1^Ser616^	Hepatocellular carcinoma cell lines (HepG2, MHCC97H, Huh7, QSG7701, QGY7703)	Mediate higher sensitivity of hepatocellular carcinoma	(97)
	SIRT3/GSTP1/JNK	Hepatocellular carcinoma cell lines (SMMC-7721, Huh-7, PLC/PRF/5)	Enhance drug sensitivity of hepatocellular carcinoma	(98)
	p53	Small-cell lung cancer cell lines (NCI-H446, NCI-H526 cells)	Induce apoptosis and increase chemosensitivity	(99)
	FOXO3α/CDT1	Lung cancer cell lines (H460, A549, HCC1588)	Enhance the sensitivity of lung cancer cells	(100)
	MnSOD	Human breast cancer cell lines (MCF7), MEFs	Direct the reprogramming of mitochondrial metabolism	(101)
Tumor-promoting	SOD2	Acute myeloid leukaemia cell lines (Kasumi‐1, MV4‐11 MOLM‐13, U937, KG‐1, THP‐1), human colorectal carcinoma cell lines (SW480, Caco2, HT29, HCT116)	Scavenge the oxidant products to promote the chemoresistance	(102, 103)
	MnSOD	Human colorectal carcinoma cell lines (SW620)	SIRT3 silencing enhances the effect of oxaliplatin	(104)
	PGC-1α	Human colorectal carcinoma cell lines (SW480, Caco2, HT29, HCT116)	Promote the chemoresistance	(103)
	UPR^mt^	Human breast cancer cell lines (MCF-7, MDA-MB-231)	Protect breast cancer cells from cisplatin	(105)

SIRT3 improves the sensitivity of cancer cells to chemotherapy to inhibit drug resistance. Chemotherapeutic agents, such as doxorubicin, cisplatin, oxaliplatin, and epirubicin, could inhibit the expression of SIRT3 in tumor cells. In hepatocellular carcinoma cells, SIRT3 sensitizes hepatocellular carcinoma cells to chemotherapy drugs *via* regulating mitochondrial cyclooxygenase-2 (mito-COX-2)/Drp1 with phosphorylation at serine 616 (p-Drp1^Ser616^) and glutathione S-transferase pi 1 (GSTP1)/c-Jun N-terminal kinase (JNK) signaling pathway (97, 98). In lung cancer, SIRT3 enhances the sensitivity of lung cancer cells to cisplatin by deacetylating mutant p53 in small-cell lung cancer and elevating the FOXO3α/chromatin licensing and DNA replication factor 1 (CDT1) axis in lung cancer (99, 100). In breast cancer, SIRT3 expression is reduced. Lowered expression of SIRT3 leads to an increase in MnSOD acetylation to direct the reprogramming of mitochondrial metabolism, thus, resulting in cisplatin and doxorubicin resistance (101). Moreover, SIRT3 enhances the sensitivity of ovarian cancer cells to cisplatin by promoting mitochondrial fission (106). Therefore, activation of SIRT3 protein may be a way to improve cancer treatment, especially to overcome the resistance of cancer cells to chemotherapy drugs.

Oppositely, SIRT3 also endows tumor cells with resistance to chemotherapy. Cancer cells exhibit enhanced ROS production and accumulation to give them an advantage in chemotherapy resistance, while ROS excess can lead to growth inhibition and apoptosis. Interestingly, SIRT3 could precisely reduce ROS production and result in cell survival (71, 107–109). When SIRT3 is highly expressed, tumor cells can balance ROS production, and become more resistant to chemotherapy drugs (71). Similarly, SIRT3 could deacetylate SOD2, and contribute to acute myeloid leukaemia chemoresistance *via* inhibiting the cytarabine-induced production of ROS to regulate mitochondrial OXPHOS (102, 103). In colorectal cancer, SIRT3 silencing not only reduces the antioxidant capacity of the cell by regulating MnSOD acetylation and increases cell death to enhance the effect of oxaliplatin but also regulates mitochondrial function under oxidative conditions through PGC-1α, thereby decreasing the chemoresistance to the colorectal cancer cells (103, 104). In this regard, SIRT3 inhibitors may be an attractive drug for the therapy of colorectal cancer in combination with cisplatin. In breast cancer, SIRT3 knockdown could enhance the effect of chemotherapy drugs. SIRT3 protects breast cancer cells from cisplatin by increasing UPR^mt^, suggesting that inhibition of SIRT3 is beneficial for enhancing chemotherapy sensitivity in breast cancer (105). SIRT3 silencing could produce an increment in ROS production and compromise the antioxidant response, thus, producing an enhancement of cisplatin-triggered apoptosis, which sensitizes breast cancer cells to cisplatin treatments (110).

In a word, the impact of SIRT3 on chemotherapy drug resistance varies with tumor type, which needs further study. It could be seen that SIRT3-mediated effects on mitochondrial metabolism reprogramming by regulating ROS levels are crucial for the chemoresistance of tumor cells. The selection of SIRT3 activators/inhibitors in combination with chemotherapeutic agents may offer a potential approach to cancer treatment to prevent the formation of a microenvironment conducive to tumor recurrence.

## Perspectives for Therapy Targeting SIRT3 in Cancer

Existing studies support the dual role of SIRT3 in cancer development, suggesting that SIRT3 activators/inhibitors can be served as cancer treatments, which is a very attractive strategy for cancer therapy. Therefore, the development and application of targeted SIRT3 as a small molecule of anti-cancer drugs has been a hotspot for many researchers. In this section, we mainly discuss the therapeutic value of SIRT3 activators/inhibitors in cancer treatment (summarized in **Table 4**).

**Table 4:** Modulators targeting SIRT3 protein in cancer.

Modulators	Compound	The mechanism of function	Effect	Cancer	References
Activators	33c (ADTL-SA1215)	SIRT3-driven autophagy/mitophagy signaling pathways	Inhibit the proliferation and migration	Breast cancer	(111)
	1-methylbenzylamino amiodarone	Induce autophagy	Induce autophagy-associated cell death	Breast cancer	(95)
	Resveratrol	Unclear	Natural anticarcinogenic agent	–	(112)
	Honokiol	Activate mitochondrial SIRT3 to suppress HIF-1α expression; protect against doxorubicin-induced cardiotoxicity	Block lung cancer cells growth; contribute to adjuvant therapy of chemotherapy	Lung cancer	(113, 114)
	Silybin	Eliminate ROS; decrease renal toxicity by cisplatin-induced	Inhibit apoptosis; contribute to adjuvant therapy of chemotherapy	–	(115)
	Ganoderic acid D	Induce the deacetylated CypD by up-regulating SIRT3	Inhibit the energy reprogramming	Colon cancer	(116)
	Melatonin	Enhance PDH activity by SIRT3 stimulation	Reverse Warburg metabolism with increased OXPHOS	Lung cancer	(117)
Inhibitors	4′‐bromo‐resveratrol	Inhibit SIRT3 to cause metabolism reprogramming	Decrease cell proliferation	Melanoma	(118)
	Cambinol analogues	Unclear	Good anti-cancer potential	–	(119)
	Analogs of N^Ɛ^-acyl-lysine	Unclear	Good anti-cancer potential	–	(120)
	YC8-02	Impair glutamine flux to the TCA cycle *via* SIRT3 depletion	Induce autophagy and cell death	Diffuse large B-cell lymphomas	(66)
	8-mercapto-3,7-dihydro-1H-purine-2,6-dione	Interact with SIRT3 through hydrophobic interactions	Anti-cancer potential	–	(121)
	EX-527	Occupy the nicotinamide site and neighboring pocket contacting NAD^+^	Anti-cancer potential	–	(122)
	LC-0296	Modulate ROS levels	Inhibit cell survival and proliferation, and promote apoptosis	Head and neck cancer	(123)
	BZD9Q1	Induce cell cycle arrest at the G2/M phase	Induct apoptosis	Oral cancer	(124)
	Butyrate	Inhibit complex and PDHA1 through SIRT3 inactivation to relieve the inhibitory phosphorylation, thereby reversing the “Warburg effect”	Create metabolic stress, promote apoptosis and inhibit the growth of cancer cells	–	(125)
	Tenovin-6	Unclear	Anti-tumor activity	Melanoma gastric cancer	(126)
	Albendazole	Induce SIRT3 degradation	Inhibit the survival of leukemia cell	Leukemia	(127)
	2-methoxyestradiol	Bind to the typical and allosteric inhibitor binding sites on SIRT3	Disturb normal mitochondrial functions	Osteosarcoma	(128)
	A671	Bind and activate SAP18 to suppress SIRT3 transcription	Potent inhibitory effect against T-cell lymphoma and erythroleukemia	T-cell lymphoma and erythroleukemia	(129)
	Linalool	Down-regulate SIRT3 to increase the acetylation of SOD2 and ROS	Inhibit the growth of cancer cells	Glioma	(130)

“-” indicates the application in cancer cells, but not specific cancers.

## Application of SIRT3 Activators in Cancer

There are few reports about SIRT3 activators in cancer treatment. Compound 33c (ADTL-SA1215) may be the earliest SIRT3-specific small molecule activator in the current research field, which can regulate the SIRT3-driven autophagy/mitophagy signaling pathways to inhibit the proliferation and invasion of human breast cancer cells (111). 1-methylbenzylamino amiodarone (MA) is another SIRT3 novel small-molecule activator that induces autophagy-associated cell death in breast cancer (95). Although few SIRT3 activators have been reported, there are many active modulators of SIRT3 that can elevate SIRT3 expression. Most SIRT3 active modulators are derived from natural products, such as Resveratrol, Honokiol, Silybin, and Ganoderic acid D (GAD). As a natural anti-cancer agent, Resveratrol activates SIRT3 to regulate cancer at different stages, but the mechanism of action of Resveratrol is still unclear (112). As a natural phenolic compound, Honokiol has anti-cancer activity. Honokiol blocks the growth of lung cancer cells by activating SIRT3 to inhibit HIF-1α expression, and also be used as adjuvant chemotherapy to prevent doxorubicin-induced cardiotoxicity in tumors transplanted mice (113, 114). Silybin is another SIRT3 activator derived from natural plants. Activation of SIRT3 can reduce ROS production and inhibit apoptosis to protect kidney cells from death, and this protection can also be used in adjuvant chemotherapy by reducing cisplatin-induced nephrotoxicity (115). GAD is the main active component of Ganoderma lucidum, and GAD inhibits the energy metabolism reprogramming of colon cancer cells by activating SIRT3 to induce deacetylation of CypD (116). In addition, Melatonin can also activate SIRT3, then the SIRT3/PDH axis is stimulated to improve the ROS production to induce apoptosis of lung cancer cells (117).

## Application of SIRT3 Inhibitors in Cancer

The development of SIRT3 inhibitors is much easier than the development of SIRT3 activators. Based on the structure and biochemical function of SIRT3, a variety of SIRT3 inhibitors have been developed and applied for cancer treatment, but it has been challenging to develop high-efficiency inhibitors with few side effects. On the one hand, the design of structural analogues based on acetylated substrates is the most common and effective design strategy. The small-molecule 4’‐bromo‐resveratrol (4’‐BR) has an inhibitory effect on SIRT3, and the reduced expression of SIRT3 could cause metabolism reprogramming, resulting in a significant anti-cancer effect in melanoma cells (118). Both Cambinol and N^Ɛ^-acyl-lysine analogs are all SIRT3 inhibitors with promising anti-cancer potential, but their anti-cancer mechanism remains unclear (119, 120). Another SIRT3-targeting inhibitor, YC8-02, induces autophagy and cell death by decreasing glutamine flux into the TCA cycle in diffuse large B-cell lymphomas cells (66). Lately, a novel SIRT3 inhibitor based on the 8-mercapto-3,7-dihydro-1H-purine-2,6-dione scaffold has been identified to inhibit SIRT3 through hydrophobic interactions, which provides new structural information for the development of effective SIRT3 inhibitors (121). On the other hand, the activity of SIRT3 could be decreased by NAD^+^ coenzyme inhibitors by inhibiting the progress of the deacetylate reaction. EX-527 has anti-cancer promise and inhibits the less active SIRT3 by holding the nicotinamide site and the adjacent pocket contacting NAD^+^ (122). LC-0296 is also considered a novel SIRT3 inhibitor because it is structurally an NAD^+^ inhibitor. LC-0296 inhibits cell survival and proliferation by regulating ROS and promoting head and neck cancer cell apoptosis (123).

Here, many other types of SIRT3 inhibitors have also been reported for cancer treatment. Compound BZD9Q1, as a SIRT3 inhibitor, elicits cytostatic effects by inducing cell cycle arrest in the G2/M phase to show promising anti-cancer properties against oral cancer (124). Butyrate acts as an inhibitor of SIRT3 to promote apoptosis of cancer cells *via* inducing high acetylation of PDHA1 to reverse the “Warburg effect” (125). Tenovin-6, a micromolar SIRT3 inhibitor, has anti-tumor ability in melanoma and gastric cancer cells, but its inhibitory effect remains unclear (126). Albendazole, an anthelmintic drug, is known as an inhibitor of leukemia cell survival by inducing SIRT3 degradation (127). 2-methoxyestradiol (2-ME) inhibits SIRT3 activity by combining with the binding site of canonical allosteric inhibitor, thereby killing osteosarcoma cells by disrupting normal mitochondrial function (128). Compound 3-O-chloroacetyl-gagamine (A671) binds and activates SIN3 Associated Protein 18 (SAP18) to inhibit the SIRT3 transcription, which has a good anti-cancer effect in T-cell lymphoma and erythroleukemia (129). Moreover, linalool inhibits the growth of glioma cells by stimulating SOD2-ROS signaling *via* SIRT3 inhibition (130).

## Role of microRNA in SIRT3 Expression and Cancer Development

MicroRNA (miRNA) is a class of short non-coding RNA that performs its functions by regulating the expression of target genes at the stage of post-transcriptional. Dysregulation of miRNA has been supported to be closely related to carcinogenesis (131). Actually, miRNA is capable of controlling SIRT3 gene transcription in tumors to affect the progression of cancer (summarized in **Table 5**).

**Table 5:** The targeting of SIRT3 protein by microRNA in cancer.

microRNA	The mechanism of function	Cancer	References
miR-1225-5p	Activate the Wnt/β-catenin pathway to improve tumor cell proliferation and metastasis	Thyroid cancer	(132)
miR-224	Inhibit AMPK and up-regulate HIF-1α to promote cell growth, angiogenesis, and metastasis	Non-small cell lung cancer	(133)
miR-494	Induce EndMT and promote the metastasis and progression by targeting the SIRT3/TGF-β/SMAD signaling pathway	Hepatocellular carcinoma	(134)
miR-31	Disrupt mitochondrial activity to promote oxidative stress	Oral carcinoma	(135)
miR-708-5p	Ablate the stimulatory activity on cell viability, invasion, and migration	Pancreatic ductal adenocarcinoma	(136)
miR-421	Inhibit Notch-1 pathway to reduce cell proliferation and invasion	Gastric cancer	(137)
miR-6858-5p	Inhibit growth-promoting AKT signaling pathway	Glioblastoma multiforme	(138)
miR-761	Modulate the response to pazopanib	Synovial sarcoma	(139)

SIRT3 is a target of miR-1225-5p, miR-224, miR-494, miR-31, miR-708-5p, miR-421, miR-6858-5p, and miR-761. Notably, downregulation of miR-1225-5p and upregulation of miR-224 may promote tumor cell growth, proliferation, and metastasis *via* targeting SIRT3 directly in thyroid cancer and non-small cell lung cancer cells (132, 133). In hepatocellular carcinoma, the miR-494 induces endothelial to mesenchymal transition (EndMT) to promote cancer metastasis by targeting the SIRT3 signaling pathway (134). Furthermore, miR-31 directly targets SIRT3 to activate oxidative stress by disrupting mitochondrial activity in oral carcinoma (135). On the contrary, miR-708-5p and miR-421 inhibit the process of pancreatic ductal adenocarcinoma and gastric cancer by targeting SIRT3 to ablate the stimulatory activity on cell viability, invasion, and migration (136, 137). In the glioblastoma multiforme, the SIRT3 expression level could be decreased by miR-6858-5p mimic, which inhibits the growth-promoting AKT signaling pathway (138). In addition, miR-761 is closely associated with SIRT3-mediated chemical resistance, and changes in miR-761 abundance targeted SIRT3 to modulate the response of synovial sarcoma cells to pazopanib (139). Therefore, altered miRNA expression affects the signaling pathway of SIRT3 involved in cancer progression. The specific connection between miRNA and SIRT3 role, and whether miRNA can be used as an indicator of SIRT3-mediated related cancer development process should be further explored, which provides a potential basis for targeting SIRT3 to find new approaches in cancer therapy.

## Discussion

Pathologic SIRT3 expression involves complex molecular signaling pathways and affects tumor metabolism, metastasis, and prognosis. As an important intracellular regulatory factor of energy metabolism, SIRT3 has the ability of metabolism reprogramming and contributes greatly to cancer fate. Available data have confirmed the dual role of SIRT3 in cancer, and the effects of SIRT3 are different at different stages of cancer development, although the study of such phenomena remained unsystematic.

Multiple studies have shown that the increase of mitochondrial ROS level is usually an important cause of the development of cancer. The role of SIRT3 in cancer is mainly to remove excess ROS through deacetylating and activating its substrates. SIRT3 regulates cell metabolism reprogramming, such as TCA, OXPHOS, and FA metabolism, to maintain mitochondria homeostasis, thereby affecting the fitness and survival of tumor cells. However, SIRT3 plays a carcinogenic role in some OXPHOS-addiction cancers, SIRT3 could promote glycolysis and tumorigenesis by maintaining ROS levels at an appropriate level, which depends on the cellular context. Interestingly, most studies have shown that SIRT3 could promote cancer metastasis, which may be closely related to the SIRT3-mediated tumor cell metabolism reprogramming and the EMT. Of course, SIRT3 can significantly inhibit cancer metastasis *via* inhibiting ROS levels, EMT, and the Src/FAK signaling pathway. Nowadays, the presence or absence of organ and tissue metastases is used as a predictor of patient survival, thus, using SIRT3 to predict cancer metastasis patient survival needs further study. Similarly, the impact of SIRT3 on cancer chemoresistance is also bidirectional. SIRT3-mediated effects on mitochondrial metabolism reprogramming by regulating ROS levels are crucial for the chemoresistance of tumor cells. Thus, the role of SIRT3 must not be generalized. The function of SIRT3 is different in different tumor tissues and normal tissues, which is related to the specificity of tumor cell type and the cellular context.

Importantly, SIRT3 is a potentially attractive molecular target based on its dual role in cancer. Several potential SIRT3 activators/inhibitors have been tested in different cell lines of cancer, but rarely *in vivo*. Further exploration of the biological function and structure of SIRT3 will contribute to the exploration of satisfactory with no side effects small-molecule modulators targeting SIRT3. However, the function of SIRT3 varies in different cancer types or at different stages of the same cancer type, which makes it relatively difficult for cancer therapy to target SIRT3. On the one hand, SIRT3 has a protective effect on most organs in the body, and inhibition of SIRT3 may affect the normal functions of other organs. On the other hand, activation of SIRT3 may also have unimaginable carcinogenic effects under certain contexts. Perhaps, combining SIRT3 activators/inhibitors with other drugs targeting a specific cancer type to design personalized therapy programs would be a potential treatment strategy. Thus, it will be a good choice to appropriately regulate SIRT3 in personalized therapy, so that it is in a dynamic balance, especially to overcome resistance to treatment.

In the future, further exploration of the dual role of SIRT3 in cancer progression should be done as follows. a) examining the role of SIRT3 in different cancer types or at different stages of the same cancer type and in both *in vivo* and *in vitro* settings; b) observing metabolism reprogramming changes that SIRT3-regulated is a driver or passenger of cancer progression; c) investigating the relationship between pathological expression of SIRT3 and specific predisposition to cancer metastasis; d) conducting of cohort studies using SIRT3 to predict cancer metastasis patient survival and establish prognosis evaluation systems; e) further developing of SIRT3 activators/inhibitors for therapeutic potential with no side effects to design personalized therapy programs. In conclusion, SIRT3 is a promising biomarker and plays an essential role in different cancer types. Exploring the function of SIRT3 in the field of cancer continues to enrich our knowledge bases. In addition, the personalized therapy combined application of SIRT3 small molecule modulators with the other drug targeting a specific cancer type has a promising future for the patients.

## Author Contributions

QZ and JZ: wrote the original draft. FL, SG, LZ, and JL: reviewed and editing. The final submitted version was supervised and approved by QQ and YS. All authors contributed to the article and the final version was approved by all of them.

## Funding

This work was supported by the Shanghai Sailing Program (No. 20YF1445800) and Shanghai Clinical Research Center for Acupuncture and Moxibustion (No. 20MC1920500).

## Conflict of Interest

The authors declare that the research was conducted in the absence of any commercial or financial relationships that could be construed as a potential conflict of interest.

## Publisher’s Note

All claims expressed in this article are solely those of the authors and do not necessarily represent those of their affiliated organizations, or those of the publisher, the editors and the reviewers. Any product that may be evaluated in this article, or claim that may be made by its manufacturer, is not guaranteed or endorsed by the publisher.
